# Associations of Proteomics With Hypertension and Systolic Blood Pressure: KORA S4/F4/FF4 and KORA Age1/Age2 Cohort Studies

**DOI:** 10.1161/HYPERTENSIONAHA.123.22614

**Published:** 2024-03-06

**Authors:** Jie-sheng Lin, Agnese Petrera, Stefanie M. Hauck, Christian L. Müller, Annette Peters, Barbara Thorand

**Affiliations:** Institute of Epidemiology (J.-s.L., A. Peters, B.T.), Helmholtz Zentrum München, German Research Center for Environmental Health (GmbH), Neuherberg, Germany.; Metabolomics and Proteomics Core (A. Petrera, S.M.H.), Helmholtz Zentrum München, German Research Center for Environmental Health (GmbH), Neuherberg, Germany.; Institute of Computational Biology (C.L.M.), Helmholtz Zentrum München, German Research Center for Environmental Health (GmbH), Neuherberg, Germany.; Institute for Medical Information Processing, Biometry, and Epidemiology (IBE), Faculty of Medicine, LMU Munich, Pettenkofer School of Public Health, Munich, Germany (J.-s.L., B.T.).; Department of Statistics (C.L.M.), Ludwig-Maximilians-Universität München, Munich, Germany.; Chair of Epidemiology, Institute for Medical Information Processing, Biometry and Epidemiology, Medical Faculty (A. Peters), Ludwig-Maximilians-Universität München, Munich, Germany.; Center for Computational Mathematics, Flatiron Institute, New York, NY (C.L.M.).; German Center for Diabetes Research, Partner München-Neuherberg, Germany (A. Peters, B.T.).

**Keywords:** blood pressure, cohort studies, hypertension, Mendelian randomization analysis, proteomics

## Abstract

**BACKGROUND:**

Hypertension, a complex condition, is primarily defined based on blood pressure readings without involving its pathophysiological mechanisms. We aimed to identify biomarkers through a proteomic approach, thereby enhancing the future definition of hypertension with insights into its molecular mechanisms.

**METHODS:**

The discovery analysis included 1560 participants, aged 55 to 74 years at baseline, from the KORA (Cooperative Health Research in the Region of Augsburg) S4/F4/FF4 cohort study, with 3332 observations over a median of 13.4 years of follow-up. Generalized estimating equations were used to estimate the associations of 233 plasma proteins with hypertension and systolic blood pressure (SBP). For validation, proteins significantly associated with hypertension or SBP in the discovery analysis were validated in the KORA Age1/Age2 cohort study (1024 participants, 1810 observations). A 2-sample Mendelian randomization analysis was conducted to infer causalities of validated proteins with SBP.

**RESULTS:**

Discovery analysis identified 49 proteins associated with hypertension and 99 associated with SBP. Validation in the KORA Age1/Age2 study replicated 7 proteins associated with hypertension and 23 associated with SBP. Three proteins, NT-proBNP (N-terminal pro-B-type natriuretic peptide), KIM1 (kidney injury molecule 1), and OPG (osteoprotegerin), consistently showed positive associations with both outcomes. Five proteins demonstrated potential causal associations with SBP in Mendelian randomization analysis, including NT-proBNP and OPG.

**CONCLUSIONS:**

We identified and validated 7 hypertension-associated and 23 SBP-associated proteins across 2 cohort studies. KIM1, NT-proBNP, and OPG demonstrated robust associations, and OPG was identified for the first time as associated with blood pressure. For NT-proBNP (protective) and OPG, causal associations with SBP were suggested.

**NOVELTY AND RELEVANCE**

**What Is New?**

Conducted both discovery and validation analyses based on 2 large prospective cohort studies.

Utilized state-of-the-art proteomic technology for the measurement of 233 inflammation and cardiovascular disease–related proteins.

**What Is Relevant?**

Discovery and validation of 7 hypertension-associated and 23 systolic blood pressure–associated proteins.

Reported robust positive associations of KIM1, NT-proBNP, and OPG with hypertension and systolic blood pressure.

Provided suggestive evidence for potential causal associations of NT-proBNP (inverse) and OPG (positive) on systolic blood pressure.

**Clinical/Pathophysiological Implications?**

Contributed valuable insights into the molecular mechanisms underlying hypertension.

Identified novel biomarkers, paving the way for a comprehensive definition and assessment of hypertension in the future.

Hypertension, characterized by persistently high blood pressure (BP), is a complex condition involving multiple pathophysiological mechanisms and target organs such as the heart, brain, and kidney. BP is regulated by a complex interplay of multiple pathophysiological mechanisms, including the sympathetic nervous system, REN (renin)-angiotensin-aldosterone system, endothelium, and immune system.^1^ High levels of BP are related to multiple adverse health outcomes, such as cardiovascular disease (CVD) and kidney disease.^2^ An estimated 1.28 billion adults aged 30 to 79 years had hypertension globally in 2019 based on the World Health Organization definition of hypertension.^3,4^ The cutoff value of BP to define hypertension is mainly based on continuous associations between a range of BP levels and CVD risks, and it has changed over time. In the American College of Cardiology/American Heart Association guidelines released in 2017, hypertension is redefined as systolic BP (SBP) ≥130 mm Hg or diastolic BP ≥80 mm Hg.^5^

Notably, hypertension is primarily defined based on a readout of BP without involving its pathophysiological mechanisms. BP can naturally fluctuate throughout the day, rising in the morning and falling in the late afternoon and evening. Hypertension is a complex condition, making it challenging and insufficient to define solely on a readout of BP or a single marker, which may oversimplify the complexity of hypertension and potentially overlook key aspects of the condition. Therefore, there is a growing need for new biomarkers that supplement the definition of hypertension, further improve the prediction of hypertension progression, and provide information on patients’ responses to treatment.

Proteomics allows for the identification of hundreds of proteins, making it a useful tool to discover new biomarkers and explore the underlying mechanisms of diseases. However, there are only a few proteomic studies about hypertension in humans. Gajjala et al^6^ compared the expression of 403 plasma proteins between 118 patients with hypertension and 85 normotensive controls and identified 27 proteins differentially expressed. Similarly, Xu et al^7^ identified 111 of 404 serum proteins differentially expressed between 20 patients with hypertension and 20 controls and found 4 proteins involved in the REN-angiotensin-aldosterone system. In a urinary proteomic study among 56 patients with hypertension and 19 controls, Matafora et al^8^ found that patients with hypertension had higher levels of urinary uromodulin, which regulates water and salt balance and BP. The close relation between BP change and age is a challenge for longitudinal proteomic studies on hypertension,^9^ and thus, previous proteomic studies in humans tend to be cross-sectional studies with small sample sizes. To date, there is only 1 longitudinal proteomic study on primary hypertension. Lin et al^10^ investigated the associations of 79 plasma CVD-related proteins with BP progression over 5 years and found that REN was positively associated with BP progression in the discovery cohort (n=804) but not in the validation cohort (n=2659). Only 2 repeated measurements of BP were included in their longitudinal analysis, with a relatively short follow-up duration.

Therefore, we aimed to assess the association of 233 plasma proteins with hypertension and SBP in a community-based prospective cohort, with a median follow-up time of 13.4 years and 2 follow-up visits. Furthermore, we validated the results in another cohort study. Additionally, we explored the potential causality of the identified associations through a 2-sample Mendelian randomization (MR) approach.

## METHODS

### Data Availability

Because of the sensitive nature of the data collected for this study and because the informed consent given by study participants does not cover data posting in public databases, cooperation partners can obtain permission to use data under the terms of a project agreement (https://helmholtz-muenchen.managed-otrs.com/external).

A full description of the methods section is available in Text S1, with a summary provided below.

### Study Population

**Figure 1.**
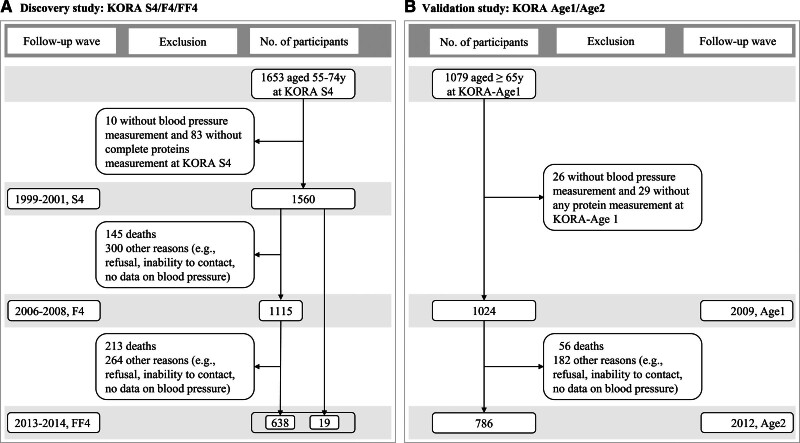
**Flowchart of study participants. A**, Participants from KORA (Cooperative Health Research in the Region of Augsburg) S4/F4/FF4 study included in discovery analysis. **B**, Participants from KORA Age1/Age2 study included in validation analysis.

The MONICA study (Monitoring of Trends and Determinants in Cardiovascular Diseases) conducted 3 health surveys S1 to S3 between 1984 and 1995 in Augsburg, Germany, and the KORA study (Cooperative Health Research in the Region of Augsburg) expanded on MONICA Augsburg by recruiting participants for a fourth survey (S4) based on the same criteria between 1999 and 2001 (Figure S1).^11^ The MONICA/KORA study was approved by the local ethical committee, and all participants provided written informed consent. The present study was based on 1653 participants aged 55 to 74 years at KORA S4 and its 2 subsequent follow-up surveys, KORA F4/FF4 (Figure 1A). A total of 1560 participants were included at KORA S4 after the exclusion of 10 participants without measurement of BP and 83 with incomplete measurement of proteins. Participants without follow-up information on BP were excluded at F4 and FF4, respectively, leaving 1115 participants at F4 and 657 participants at FF4 (19 participants were only followed up at FF4, but not at F4). In summary, 1560 participants with 3332 observations from KORA S4/F4/FF4 were included for discovery analysis, with a median follow-up time of 13.4 (25th percentile, 7.1; 75th percentile, 13.5) years.

For validation, a subset of participants was drawn from the KORA Age1/Age2 study (Figure S1), which included participants at MONICA/KORA S1 to S4 born in the year 1943 or before (ie, age ≥65 years). In 2009 (KORA-Age1), a random subsample of 1079 participants underwent medical examinations and were invited to participate in the follow-up in 2012 (KORA-Age2). Figure 1B shows that 1024 participants with measurements on BP and proteins at KORA-Age1 and 786 participants with follow-up information at KORA-Age2 were included in the validation analysis, with a median follow-up time of 2.87 (25th percentile, 2.79; 75th percentile, 2.94) years. Since the KORA Age1/Age2 study also included participants at KORA S4, 142 of the 1024 participants overlapped with the 1560 participants from KORA S4/F4/FF4, but examinations were performed at different time points.

### Assessment of Proteins, BP, and Covariates

Detailed assessment methods are available in Text S1. Olink proximity extension assay technology^12^ was used to measure plasma proteins, including CVD II, CVD III, and inflammation panels. At KORA S4, 233 proteins were measured, and of these, 231 proteins were available at KORA-Age1 (Table S1). *Z*-score transformations were conducted for all proteins. BP and covariates such as age and smoking status were measured at baseline and follow-up. Hypertension was defined based on the World Health Organization definition.^3^

### Statistical Analysis

#### Discovery Analysis in KORA S4/F4/FF4

To address bias from participant dropouts (Figure 1), we calculated inverse probability weights^13^ and applied these in the following analyses. Generalized estimating equations were used to estimate the associations of proteins with repeated measurements of dichotomous hypertension (yes/no) and continuous SBP using the R package geepack. Any participant with protein measurement and BP data for at least 1 time point was included.

In the discovery analysis of the KORA S4/F4/FF4 study, the associations of 233 proteins with prevalent hypertension and levels of SBP were estimated by generalized estimating equations applying 2 models. Model 1, adjusted for age and sex; and model 2, model 1 plus body mass index, smoking status, alcohol consumption, physical activity, naturally log-transformed triglycerides, high-density lipoprotein cholesterol, use of lipid-lowering medication, prevalent diabetes, prevalent CVD, fasting status, and kidney function. For the associations with SBP, both models were further adjusted for the use of antihypertensive medication. Covariates in both models were treated as time-varying covariates, except sex. The Benjamini-Hochberg false discovery rate (FDR) was used to adjust for multiple testing, and FDR <0.05 was considered statistically significant.^14^

#### Validation Analysis in KORA Age1/Age2

The proteins significantly associated with hypertension or SBP (FDR <0.05) in the discovery analysis were taken to validate their associations with hypertension or SBP in the KORA Age1/Age2 study using generalized estimating equations, applying the same model 2 as described above, respectively. Proteins were considered validated if they demonstrated significant associations at a threshold of *P*<0.05.

#### Sensitivity Analysis

Sensitivity analyses were performed as follows: (1) in the validation analysis, a sensitivity analysis was conducted after excluding 142 participants who overlapped with KORA S4/F4/FF4; and (2) for associations with SBP, linear mixed-effects models were used in both discovery and validation analyses, applying the aforementioned model 2, using R package lme4.

#### MR Analysis on SBP

A 2-sample MR analysis was conducted to estimate the potential causal associations of proteins with SBP using publicly available genome-wide association studies. Single-nucleotide polymorphisms (SNPs), serving as instrumental variables for proteins, were selected from a genome-wide association study mapping protein quantitative trait loci in 35 571 European-ancestry people.^15^ Of 27 validated proteins, 26 had available protein quantitative trait loci based on *cis*-SNPs with *P*<5×10^−8^. To refine SNPs, linkage disequilibrium clumping (r^2^<0.01 within a 10 000-kb region) was applied based on a reference panel using 1000 Genomes data from 503 European samples.^16^ The associations of SNPs with SBP were extracted from a genome-wide association study identifying loci associated with BP in >1 million European-ancestry people.^17^ Finally, 26 proteins with 1 to 17 SNPs were used for MR analysis (Table S2) using the R package TwoSampleMR. The function MendelianRandomization::mr_mr_ivw was used to evaluate bias due to participant overlap.^18,19^ Details are presented in Text S1.

## RESULTS

### Characteristics of the Study Population

The characteristics of participants at KORA S4 and KORA-Age1 (ie, baseline for each study) are presented in the Table. In KORA S4, participants had a significantly lower mean age of 63.9 (5.46) years compared with 75.9 (6.57) years in KORA-Age1. Unexpectedly, despite the age difference, the average SBP in KORA S4 was only slightly lower at 136.4 (20.5) mm Hg compared with 138.6 (21.0) mm Hg in KORA-Age1. The difference in the proportion of use of antihypertensive medication (36.7% in KORA S4 versus 70.1% in KORA-Age1) may partly explain this relatively small difference. Significant differences were also observed across various lifestyle- and health-related variables, as well as the prevalence of diseases. Table S3 provides detailed information on the characteristics of participants across KORA S4/F4/FF4 and KORA Age1/Age2, and characteristics for participants with and without follow-up information on BP are summarized in Table S4.

**Table.**
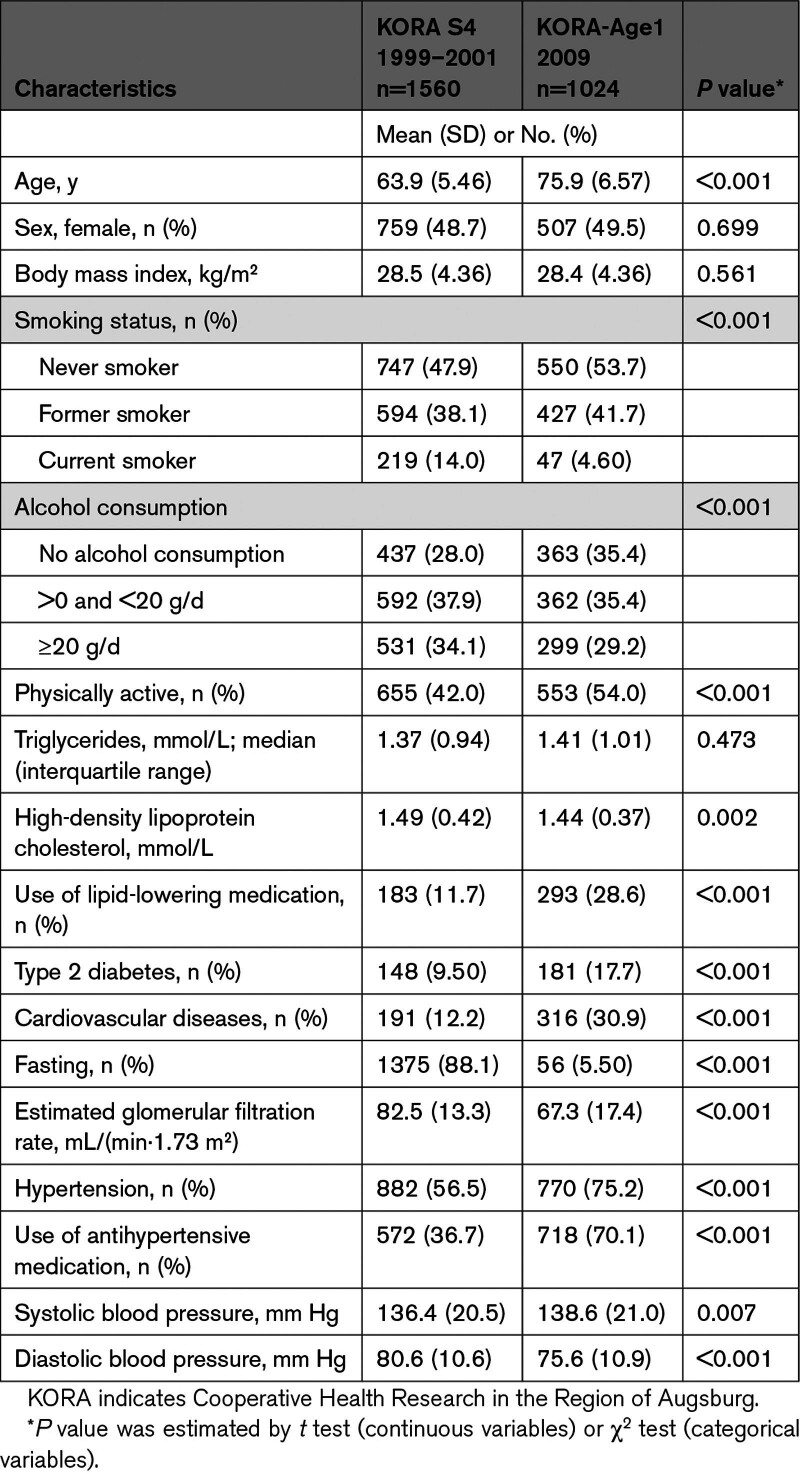
Baseline Characteristics of Participants

### Discovery of Proteins Associated With Hypertension or SBP

**Figure 2.**
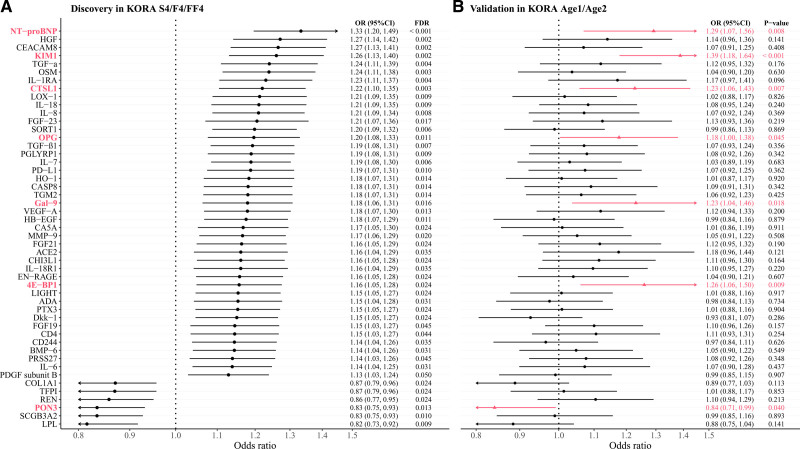
**Associations of 49 proteins with hypertension in the discovery and validation studies. A**, Significant associations of 49 proteins with hypertension in the KORA (Cooperative Health Research in the Region of Augsburg) S4/F4/FF4 study (Benjamini-Hochberg false discovery rate [FDR] <0.05). **B**, Validation of the associations of 49 proteins with hypertension in the KORA Age1/Age2 study. Proteins were considered validated at a threshold of *P*<0.05. This figure illustrates the results of model 2, as detailed in Table S9. Proteins in bold and red are successfully validated. OR indicates odds ratio. Full names of the proteins can be found in Table S1.

**Figure 3.**
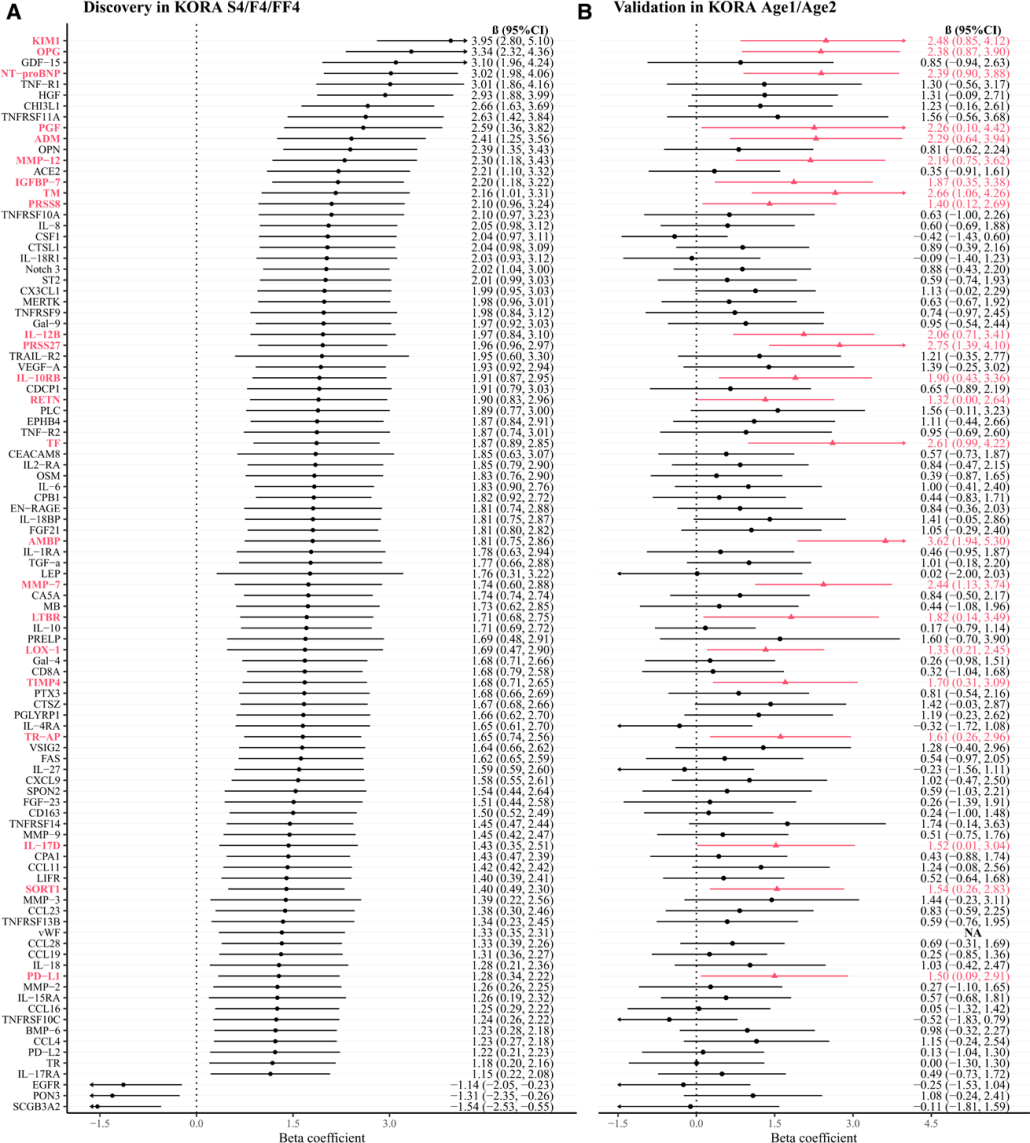
**Associations of 99 proteins with systolic blood pressure (SBP) in the discovery and validation studies. A**, Significant associations of 99 proteins with SBP in the KORA (Cooperative Health Research in the Region of Augsburg) S4/F4/FF4 study (Benjamini-Hochberg false discovery rate <0.05). **B**, Validation of the associations of 99 proteins with SBP in the KORA Age1/Age2 study. Proteins were considered validated at a threshold of *P*<0.05. This figure illustrates the results of model 2, as detailed in Table S10. Proteins in bold and red are successfully validated. NA indicates not available. Full names of the proteins can be found in Table S1.

In the discovery analyses of the associations of 233 proteins with hypertension conducted within the KORA S4/F4/FF4 study, 149 proteins were significant in model 1 (FDR <0.05), while 48 proteins remained significant after adjustment for additional covariates in model 2, and in addition, REN became significant in model 2 (Table S5; Figure 2A). Among the 49 significant proteins in model 2, 43 proteins were positively (odds ratios [ORs], 1.13–1.33) and 6 proteins were inversely (ORs, 0.82–0.87) associated with prevalent hypertension. When investigating the associations with SBP, 99 of 233 proteins were significant in model 2 (FDR <0.05), with 96 proteins positively associated (β-coefficients, 1.15–3.95 mm Hg) and 3 proteins inversely associated (β, −1.54 to −1.14) with SBP (Table S6; Figure 3A). In the sensitivity analysis for SBP using linear mixed-effects models, all 99 proteins remained significant (FDR <0.05), and an additional 18 proteins showed significance (Table S7).

**Figure 4.**
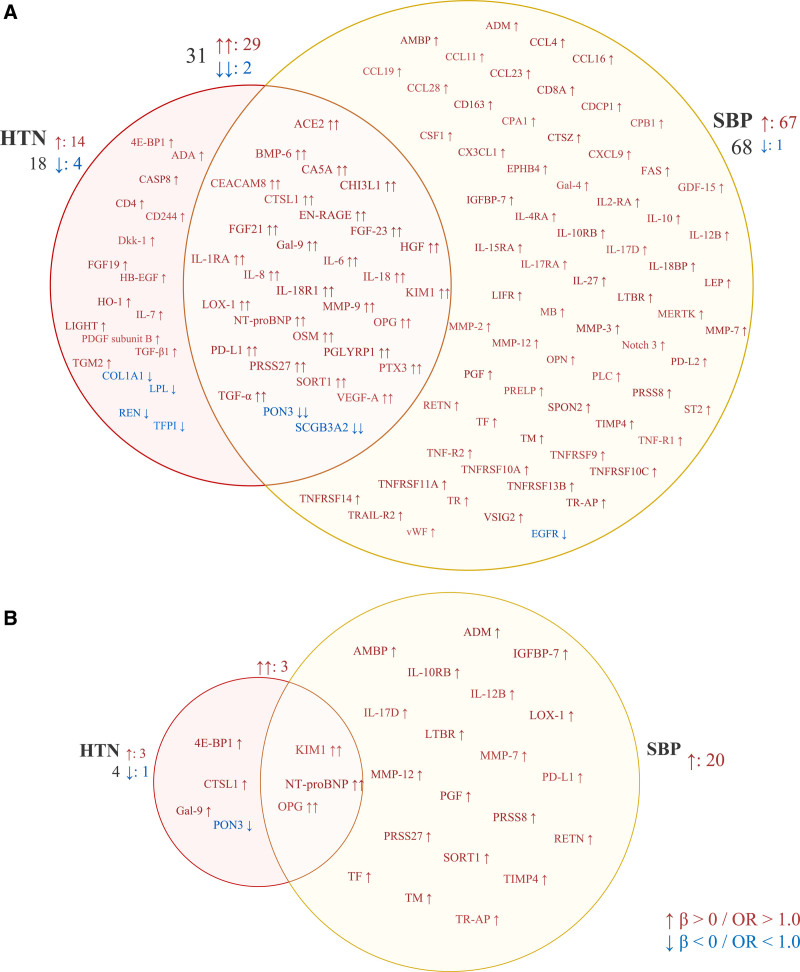
**Overlap of proteins associated with hypertension (HTN) and systolic blood pressure (SBP). A**, Overlap of proteins associated with HTN and SBP in the discovery analysis in the KORA (Cooperative Health Research in the Region of Augsburg) S4/F4/FF4 study. Detailed results are presented in Table S8. **B**, Overlap of proteins associated with HTN and SBP in the validation analysis in the KORA Age1/Age2 study. Detailed results are presented in Tables S9 and S10. OR indicates odds ratio. Full names of the proteins can be found in Table S1.

There were 31 proteins significantly associated with both hypertension and SBP (Table S8; Figure 4A). Among these 31 proteins, the top 5 proteins with the highest ORs for the associations with hypertension were NT-proBNP (N-terminal pro-B-type natriuretic peptide; OR, 1.33 [95% CI, 1.20–1.49]), HGF (hepatocyte growth factor; OR, 1.27 [95% CI, 1.14–1.42]), CEACAM8 (carcinoembryonic antigen-related cell adhesion molecule 8; OR, 1.27 [95% CI, 1.13–1.41]), KIM1 (kidney injury molecule 1; OR, 1.26 [95% CI, 1.13–1.40]), and TGF-α (transforming growth factor alpha; OR, 1.24 [95% CI, 1.11–1.39]). The top 5 proteins with the highest β-coefficients for the associations with SBP were KIM1 (β, 3.95 [95% CI, 2.80–5.10]), OPG (osteoprotegerin; β, 3.34 [95% CI, 2.32–4.36]), NT-proBNP (β, 3.02 [95% CI, 1.96–4.24]), HGF (β, 2.93 [95% CI, 1.88–3.99]), and CHI3L1 (chitinase-3-like 1; β, 2.66 [95% CI, 1.63–3.69]).

### Validation of Proteins Associated With Hypertension or SBP

When validating the 49 proteins significantly associated with hypertension in the discovery analysis, 7 proteins were associated with hypertension at a threshold of *P*<0.05 in the KORA Age1/Age2 study (Table S9; Figure 2B). Of the 99 SBP-associated proteins in the discovery study, 23 proteins were validated (Table S10; Figure 3B), and 3 validated proteins (ie, NT-proBNP, KIM1, and OPG) were associated with both hypertension and SBP (Figure 4B). Among these 27 validated proteins, 26 were positively associated with hypertension or SBP, while PON3 (paraoxonase) demonstrated an inverse association. The correlations between these 27 validated proteins are presented in Figures S2 and S3. In the sensitivity analysis (Tables S9 and S10) that excluded 142 overlapping participants, PON3 and OPG were not significantly associated with hypertension, but the direction of the associations remained consistent, indicating that the lack of significance could be due to reduced statistical power. OPG maintained its association with SBP. The sensitivity analysis using linear mixed-effects models identified 29 significant proteins (*P*<0.05), of which 21 overlapped with the above 23 SBP-validated proteins (Table S11). The overall robustness of the results was maintained.

### MR Analysis on SBP

Table S12 presents the main results of the MR analysis, including heterogeneity, pleiotropy, and Steiger directionality tests. Since no evidence of directional horizontal pleiotropy (Egger *P* value >0.05) was observed, for proteins with ≥2 SNPs, MR results from inverse variance weighted or weighted median (when heterogeneity test was significant with Q *P* value <0.05) are presented. There were 5 of 26 proteins that demonstrated potential causal associations with SBP (*P*<0.05/26). NT-proBNP (β, −2.46 [95% CI, −2.77 to −2.15) and IL-10RB (interleukin-10 receptor subunit beta; β, −0.19 [95% CI, −0.31 to −0.08]) showed inverse associations, while TIMP4 (tissue inhibitor of metalloproteinase inhibitor 4; β, 0.56 [95% CI, 0.25–0.87]), PD-L1 (programmed cell death 1 ligand 1; β, 0.28 [95% CI, 0.12–0.44]), and OPG (β, 0.41 [95% CI, 0.16–0.66]) exhibited positive associations. The Steiger directionality tests indicate that the variance explained in proteins was significantly higher than in SBP, suggesting causal directions from proteins to SBP. When accounting for potential bias due to participant overlap in the 2 genome-wide association studies, the causal associations with SBP remained robust for 4 of the 5 identified proteins, that is, NT-proBNP, OPG, PD-L1, and TIMP4 (Table S13).

## DISCUSSION

In this prospective study conducted on 2 cohorts, we identified 49 hypertension-associated and 99 SBP-associated proteins of a total of 233 CVD- and inflammation-related plasma proteins in the KORA S4/F4/FF4 study. Upon validating these proteins in the KORA Age1/Age2 study, 7 and 23 proteins were associated with hypertension and SBP, respectively. Three proteins, NT-proBNP, KIM1, and OPG, were consistently associated with both hypertension and SBP in the discovery and validation analyses. In MR analysis, 5 proteins showed potential causal associations with SBP, including IL-10RB, NT-proBNP, OPG, PD-L1, and TIMP4.

Studies about proteomics and BP in humans are limited. We only found 3 cross-sectional studies with small sample sizes^6–8^ and 1 longitudinal study.^10^ A few previously reported proteins in the 3 cross-sectional studies were replicated in our discovery analysis, including latency-associated peptide TGF-β1 (transforming growth factor beta-1),^7^ TNFRSF14 (tumor necrosis factor receptor superfamily member 14), and PLC (perlecan).^8^ Using the same protein measurement technology as in the present study, Lin et al^10^ identified REN from 79 plasma CVD I–based proteins, showing a positive association with BP progression in the discovery cohort only. In contrast, we observed an inverse association of REN with hypertension in the discovery analysis.

REN is an enzyme produced by special cells in the kidneys in response to triggers such as low blood volume, decreased sodium levels, or reduced BP.^1,20^ The release of REN initiates the REN-angiotensin-aldosterone system, which regulates blood volume and vascular resistance by controlling sodium and water retention, as well as vascular tone, ultimately leading to increases in BP.^1,20^ Low-REN hypertension, a subtype of hypertension, is characterized by low levels of REN, and this subtype accounts for nearly one-third of all patients with hypertension, while medium- or high-REN hypertension represents more than one-third.^21^ Interestingly, although Lin et al and our studies observed opposite significant REN-hypertension associations in discovery analysis, neither study was able to validate the association of REN. The different subtypes of hypertension with varying REN levels among the included participants may explain the discrepancy.

The successful replication of previously reported proteins supports the viability of the proteomic approach for identifying biomarkers for hypertension and BP. Furthermore, our discovery analysis identified several proteins associated with both hypertension and SBP that are established biomarkers for hypertension, such as IL-6 (interleukin-6), LOX-1 (lectin-like oxidized low-density lipoprotein receptor 1), NT-proBNP, TNF-α (tumor necrosis factor-alpha), and VEGF-A (vascular endothelial growth factor A).^22,23^ Additionally, NT-proBNP was successfully validated for its associations with both hypertension and SBP in our validation analysis.

NT-proBNP is an inactive peptide released along with the active peptide BNP (B-type natriuretic peptide) in a 1:1 ratio from the heart upon myocardial stretching or pressure overload on the heart. Both NT-proBNP and BNP are strongly associated with various adverse CVD outcomes and are used for the diagnosis or exclusion of heart failure.^24^ Since NT-proBNP is more stable, it forms a good marker of BNP output. BNP can regulate BP through its natriuretic, diuretic, and vasodilatory effects, which reduce sodium and water retention and ease blood vessels, resulting in decreasing blood volume and vascular resistance.^25^ In our discovery analysis, NT-proBNP was the biomarker with the strongest association with hypertension and one of the top SBP-associated biomarkers. Our results were in line with results from an American cohort study, consisting of 3798 middle-aged participants, which reported a positive association of baseline NT-proBNP with incident hypertension.^26^ However, a similar prospective study, comprising 1323 participants aged ≥45 years, failed to observe a significant association of baseline NT-proBNP with incident hypertension.^27^ A European population-based study observed that higher levels of NT-proBNP were associated with prevalent hypertension (n=5307), whereas lower baseline NT-proBNP was associated with incident hypertension n=2389).^28^ In a study using an MR approach to investigate the causal associations of 227 proteins with BP, NT-proBNP was inversely associated with both SBP and diastolic BP, but the epidemiological association analyses showed positive cross-sectional associations of NT-proBNP with BP.^29^ This aligns with the results from the present study, where we observed a positive association of NT-proBNP with hypertension and SBP, while our MR analysis found evidence for an inverse causal association of NT-proBNP with SBP. A potential explanation for the opposite direction of association between observational studies and MR could be that compensatory mechanisms and environmental factors influence protein expression,^30^ leading to elevated levels of NT-proBNP/BNP in individuals with hypertension to counteract increases in BP levels. Therefore, high NT-proBNP/BNP levels might be a consequence of BP elevation, suggesting that NT-proBNP could serve as a marker for hypertension and elevated BP.

KIM1 is a protein expressed in response to kidney injury and is positively associated with impaired kidney function.^31^ In our previous study investigating the association of proteomics with kidney function decline, we found KIM1 was the protein with the strongest positive association.^32^ While KIM1 is recognized as a key biomarker for kidney function, its direct association with BP is not as well-documented. Two cohort studies have reported that urinary KIM1 was not associated with incident hypertension.^33,34^ In contrast, our present study observed strong positive associations of plasma KIM1 with SBP and hypertension, which is consistent with several cross-sectional studies that have demonstrated positive associations of plasma/serum KIM1 with BP and hypertension.^35–37^ The difference in biosamples used to measure KIM1 may partially explain these distinct findings. As KIM1 is mainly expressed in renal tubular epithelial cells, urinary KIM1 may more directly reflect kidney damage but may not directly relate to BP change, while plasma/serum KIM1 may represent a broader systemic influence, such as inflammatory response and endothelial dysfunction, which are related to BP regulation.^1,38^ The kidneys play a key role in the regulation of BP through the REN-angiotensin-aldosterone system, and conversely, BP can also affect kidney function. Chronic high BP can damage kidney function, impacting the kidneys’ ability to effectively regulate BP.^39^ Given the evidence from observational studies and the bidirectional relationship between the kidneys and BP, KIM1 may serve as a potential marker for BP change and provide valuable insights into the complex interplay between kidney function and BP. Further studies are warranted to explore the clinical implications of KIM1 in the prediction of the development of hypertension.

OPG was another protein that demonstrated positive associations with SBP and hypertension, with the MR analysis also suggesting a positive causal association of OPG with SBP. OPG is a decoy receptor in the TNF-related activation-induced cytokine (RANKL)/TNF receptor superfamily member 11A (RANK)/OPG system, inhibiting bone resorption by obstructing the interaction between RANKL and RANK, thereby playing a key role in bone remodeling.^40^ Moreover, increasing evidence confirms the relationship of OPG with various CVD, where OPG plays a role in regulating vascular endothelial cell function and the atherosclerotic process in the arteries,^40^ which could potentially influence BP regulation. In addition to CVD, previous studies have also reported associations of OPG with kidney function.^41,42^ However, despite the close relations of OPG with CVD and kidney function, as well as the above potential mechanisms, it is important to note that direct biological and epidemiological evidence linking OPG to BP is currently lacking. Thus, more comprehensive studies are needed to fully elucidate the role of OPG in BP regulation and hypertension.

In MR analysis, we also found suggestive evidence for potential causal associations of IL-10RB, PD-L1, and TIMP4 with SBP. IL-10RB, a key component in the IL-10 signaling pathway, and PD-L1, a pivotal regulator in immune checkpoint modulation, have crucial roles in immune system regulation.^43,44^ While direct associations of IL-10RB and PD-L1 with BP are scarce, considering the known involvement of the immune system in BP regulation, it is plausible that IL-10RB and PD-L1 may influence BP. Similarly, TIMP4, a key regulator of matrix metalloproteinases, is involved in immune and inflammatory responses.^45^ TIMP4 contributes to pathological changes in the blood vessels through processes such as tissue remodeling, angiogenesis, and inflammation,^45^ suggesting that TIMP4 may also be associated with BP regulation.

Key strengths of our study include conducting both discovery and validation analyses based on 2 large prospective cohort studies and simultaneous measurement of numerous proteins. There are also several limitations to consider. First, we did not investigate the association with incident hypertension given that our participants were relatively old and exhibited a high prevalence of hypertension at baseline. Second, we were unable to account for the effect of changes in proteins during follow-up since we only measured proteins at baseline. Third, the validation study may not be perfect for validation due to differences in participant characteristics between the discovery and validation studies. Fourth, we only included 233 CVD- and inflammation-related proteins. Finally, an unexpected decline in average SBP across KORA S4/F4/FF4 was observed, probably because individuals who participated in the follow-up examinations tended to be healthier and the proportion of antihypertensive medication use increased. Although we applied inverse probability weighting, bias resulting from loss to follow-up may not be fully controlled.

In conclusion, our study identified and validated 7 hypertension-associated and 23 SBP-associated proteins across 2 cohort studies. Among these, 3 proteins—KIM1, NT-proBNP, and OPG—demonstrated robust positive associations with both hypertension and SBP. Notably, this is the first epidemiological study to report associations of OPG with hypertension and SBP. Additionally, results from the MR analysis provide evidence for a potential protective effect of NT-proBNP and a causal effect of OPG on SBP. These findings may contribute valuable insights into the molecular mechanisms underlying hypertension and provide evidence for a comprehensive definition and assessment of hypertension in the future.

## PERSPECTIVES

Our study takes a significant step forward in hypertension research by using a proteomic approach to identify biomarkers associated with hypertension and SBP. The successful discovery and validation of 7 hypertension-associated and 23 SBP-associated proteins across 2 cohort studies supports the feasibility of this approach and shows its potential for identifying novel biomarkers. Among these proteins, KIM1, NT-proBNP, and OPG demonstrated robust positive associations with both hypertension and SBP. Notably, this is the first epidemiological study to report associations of OPG with hypertension and SBP. The potential protective effect of NT-proBNP and a causal effect of OPG on SBP, as evidenced by our MR analysis, further support the robustness and credibility of our findings. Our study may enhance the understanding of the molecular mechanisms underlying hypertension and pave the way for a more comprehensive assessment of hypertension that goes beyond mere BP readings. Future studies are expected to explore these proteins as potential therapeutic targets and monitoring tools for tracking treatment responses in patients.

## ARTICLE INFORMATION

### Acknowledgments

The authors thank all participants for their long-term commitment to the KORA study (Cooperative Health Research in the Region of Augsburg), the staff for data collection and research data management, and the members of the KORA study group (https://www.helmholtz-munich.de/en/epi/cohort/kora) who are responsible for the design and conduct of the study. J.-s. Lin would like to thank the China Scholarship Council for the financial support (No. 202008440343).

### Author Contributions

J.-s. Lin drafted the analysis plan, performed the statistical analysis, interpreted the data, and wrote the first draft of the manuscript. C.L. Müller and A. Peters contributed to the analysis plan and data interpretation. B. Thorand designed the study and contributed to the analysis plan and data interpretation. A. Petrera, S.M. Hauck, A. Peters, and B. Thorand contributed data. All authors read and approved the final manuscript. J.-s. Lin and B. Thorand had primary responsibility for the final content.

### Sources of Funding

The KORA study (Cooperative Health Research in the Region of Augsburg) was initiated and financed by the Helmholtz Zentrum München, German Research Center for Environmental Health, which is funded by the German Federal Ministry of Education and Research and by the State of Bavaria. Data collection in the KORA study is done in cooperation with the University Hospital of Augsburg. Proteomics measurements were supported by the Helmholtz Institute for Metabolic, Obesity and Vascular Research, Project Initiative 2018. The funder had no role in study design, data collection, analysis, decision to publish, or preparation of the manuscript.

### Disclosures

None.

### Supplemental Material

Text S1

Figures S1–S3

Tables S1–S13

## Supplementary Material


