# Supplementary material for: Associations of Proteomics With Hypertension and Systolic Blood Pressure: KORA S4/F4/FF4 and KORA Age1/Age2 Cohort Studies
Source: Hypertension. 2024 Mar 6;81(5):1156–66. doi: 10.1161/HYPERTENSIONAHA.123.22614 (PMC11025610; doi:10.1161/HYPERTENSIONAHA.123.22614)

Supplemental Material 1: texts and figures

Associations of proteomics with hypertension and systolic blood pressure: KORA S4/F4/FF4 and KORA Age1/Age2 cohort studies

Jie-sheng Lin, MSc, Agnese Petrera, PhD, Stefanie M. Hauck, PhD, Christian L. Müller, PhD, Annette Peters, PhD, Barbara Thorand, PhD [1, 2, 3 *]

[*] **Corresponding author's contact information**

Prof. Dr. Barbara Thorand

E-mail: barbara.thorand@helmholtz-munich.de

Institution:

[1] *Institute of Epidemiology, Helmholtz Zentrum München, German Research Center for Environmental Health (GmbH), Neuherberg, Germany*

[2] *Institute for Medical Information Processing, Biometry, and Epidemiology (IBE), Faculty of Medicine, LMU Munich, Pettenkofer School of Public Health, Munich, Germany*

[3] *German Center for Diabetes Research (DZD), Partner München-Neuherberg, Neuherberg, Germany*

Text S1. Full description of the methods section

Study population

The Monitoring of Trends and Determinants in Cardiovascular Diseases (MONICA) study conducted 3 health surveys S1 to S3 among the population aged 25-74 years between 1984 and 1995 in Augsburg, Germany, and the Cooperative Health Research in the Region of Augsburg (KORA) study expanded on MONICA Augsburg by recruiting participants for a fourth survey (S4) based on the same criteria between 1999 and 2001 (Figure S1) [1]. The MONICA/KORA study was approved by the local ethical committee and all participants provided written informed consent. The present study was based on 1653 participants aged 55-74 years at KORA S4 and its two subsequent follow-up surveys KORA F4/FF4 (Figure 1A). A total of 1560 participants were included at KORA S4 after exclusion of 10 participants without measurement of BP and 83 with incomplete measurement of proteins. Participants without follow-up information on BP were excluded at F4 and FF4, respectively, leaving 1115 participants at F4 and 657 participants at FF4 (19 participants were only followed up at FF4, but not at F4). In summary, 1560 participants with 3332 observations from KORA S4/F4/FF4 were included for discovery analysis, with a median follow-up time of 13.4 (25th percentile: 7.1, 75th percentile: 13.5) years.

For validation, a subset of participants was drawn from the KORA Age1/Age2 study (Figure S1), which included participants at MONICA/KORA S1-S4 born in the year 1943 or before (i.e., age ≥ 65 years). In 2009 (KORA-Age1), a random sub-sample of 1079 participants underwent medical examinations and participated in the follow-up in 2012 (KORA-Age2). Figure 1B shows that 1024 participants with measurements on BP and proteins at KORA-Age1 and 786 participants with follow-up information at KORA-Age2 were included in the validation analysis, with a median follow-up time of 2.87 (25th percentile: 2.79, 75th percentile: 2.94) years. Since the KORA Age1/Age2 study also included participants at KORA S4, 142 out of the 1024 participants overlapped with the 1560 participants from KORA S4/F4/FF4 but examinations were performed at different time points.

Measurement of proteins

Olink's proximity extension assay technology [2] was used to measure plasma proteins at both KORA S4 and KORA-Age1, including the Proseek Multiplex cardiovascular disease (CVD) II, CVD III, and Inflammation panels (Olink, Upsala, Sweden). Normalized protein expression values on a log2 scale were generated from the measurements, where higher values represent higher protein levels. Details of the measurement and selection of the proteins have been reported previously [3]. At KORA S4, 233 proteins were measured among 1560 participants, and out of these, 231 proteins were available at KORA-Age1 among 1024 participants (Supplemental Material 2: Table S1). Z-score transformations were conducted for all proteins in participants at KORA S4 and KORA-Age1, respectively.

Assessment of blood pressure and antihypertensive medication

Information on sociodemographic characteristics, lifestyle factors, medical history, medication use, blood pressure (BP), anthropometric indices, etc. were gathered by trained medical staff during a standardized face-to-face interview or medical examination [1]. For BP measurement, participants sat in a chair with back supported for at least 5 minutes before the 1st measurement, with right lower arm resting on table at heart level. BP was measured by an automatic digital oscillometer (HEM 705CP-II Omron Corporation Japan) 3 times in a 3-minute interval. The mean of the 2nd and 3rd measurements was used for the present study. Information on medications used within the past 7 days before the interview was provided by the participants and antihypertensive medications were classified by guidelines of the German Hypertension Society. Hypertension was defined as systolic BP (SBP) ≥ 140 mm Hg and/or diastolic BP (DBP) ≥ 90 mm Hg, or reported use of antihypertensive medications given that participants were aware of having hypertension [4].

Covariates

Covariates included age, sex, body mass index, smoking status, alcohol consumption, physical activity, triglycerides, high-density lipoprotein cholesterol, use of lipid-lowering medication, use of antihypertensive medication, prevalent diabetes, prevalent cardiovascular diseases, fasting status (whether fasting for at least 8 hours before blood draw), and kidney function (creatinine-based estimated glomerular filtration rate) at baseline and follow-up. Detailed categories of categorical variables are presented in Table 1 and details of covariates measurements have been reported previously [3].

Statistical analysis

Continues variables were reported as mean ± standard deviation for normally distributed data and as median (interquartile range) for skewed data. Categorical variables were presented as total numbers with the corresponding percentages.

Discovery of hypertension and SBP-associated proteins in KORA S4/F4/FF4

To address bias from participant dropouts (Figure 1), we utilized the inverse probability weighting method [5]. The probability of each participant's dropout (yes/no) was determined using logistic regression, considering baseline covariates from model 2 below as predictors. The weight was calculated as 1/ (1-dropout probability) and applied in the following analyses. To take into account repeated measurements of hypertension and SBP for each participant, generalized estimating equations (GEE) with exchangeable correlation structure were used to estimate the associations of proteins with dichotomous hypertension (yes/no) and continuous SBP, using R package "geepack". Given the well-documented phenomenon that DBP declines after the age of 50, and hypertension in adults over 50 years is predominantly characterized by

elevations in SBP, we focus on SBP as a continuous BP outcome given the age range of our participants. Any participant with protein measurement and BP data for at least one time point was included.

In the discovery analysis among KORA S4/F4/FF4 study, the associations of 233 proteins with prevalent hypertension and levels of SBP were estimated by GEE. Two models were constructed to control potential confounders: model 1, adjusted for age and sex; model 2, model 1 plus body mass index, smoking status, alcohol consumption, physical activity, naturally log-transformed triglycerides, high-density lipoprotein cholesterol, use of lipid-lowering medication, prevalent diabetes, prevalent cardiovascular diseases, fasting status, and kidney function. For the associations with continuous SBP, both models were further adjusted for use of antihypertensive medication. Covariates in both models were treated as time-varying covariates, except sex. Benjamini–Hochberg False-discovery rate (FDR) was used to adjust for multiple testing and FDR < 0.05 was considered statistically significant [6].

Validation of hypertension and SBP-associated proteins in KORA Age1/Age2

The proteins significantly associated with dichotomous hypertension or continuous SBP (FDR < 0.05) in the discovery analysis among KORA S4/F4/FF4 study were taken to validate their associations with hypertension or SBP in KORA Age1/Age2 study using GEE, applying the same model 2 as described above, respectively. Proteins were considered validated if they demonstrated significant associations at a threshold of P-value < 0.05.

Sensitivity analyses were performed as follows: 1) In the validation analysis, sensitivity analysis was conducted after excluding 142 participants who overlapped with KORA S4/F4/FF4. 2) For associations with SBP, linear mixed-effects models were used in both discovery and validation analyses, applying the aforementioned model 2, using R package “lme4”.

Mendelian randomization analysis on SBP

Two-sample Mendelian randomization (MR) analysis was conducted to estimate the potential causal associations of proteins with SBP utilizing publicly available genome-wide association studies (GWAS). We focused the MR on SBP given its nature as a continuous variable, providing more statistical power, and benefitting from the larger scale of available GWAS compared to the dichotomous outcome of hypertension. Single nucleotide polymorphisms (SNPs), serving as instrumental variables (IVs) for proteins, were selected from a GWAS mapping protein quantitative trait loci (pQTL) of 2923 plasma proteins based on Olink’s proximity extension assay technology in 35571 participants from a European-ancestry population from the UK Biobank Pharma Proteomics Project [7]. Out of 27 validated proteins (Figure 4B), 26 had available pQTLs based on cis-SNPs with genome-wide significance (P-value < 5E-8). To refine SNPs, linkage disequilibrium clumping was applied based on a reference panel using 1000 genomes

data from 503 European samples [8], excluding SNPs with $r^2 < 0.01$ within a 10000kb region, leading to the removal of 68 to 2701 SNPs per protein. The associations of SNPs with SBP were extracted from a GWAS identifying loci associated with BP in more than 1 million European-ancestry people [9]. After excluding 84 SNPs without SNPs-SBP associations, data harmonization was performed to ensure that the effects of SNPs on proteins and SBP were corresponding to the same allele, and 2 palindromic SNPs were further excluded (Table S2), leaving 26 proteins with 1 to 17 SNPs for MR analysis.

Inverse variance weighted MR was performed for proteins with 2 or more SNPs, while Wald ratio was calculated when only 1 SNP instrument was available. Cochran's Q test was performed to test for instrument heterogeneity for proteins with 2 or more SNPs, and MR-Egger regression was used to assess directional horizontal pleiotropy for proteins with 3 or more SNPs. Weighted median MR was performed to estimate the proteins-SBP associations when the heterogeneity test was significant but without horizontal pleiotropy, whereas MR-Egger regression was used when the horizontal pleiotropy test was significant. MR Steiger directionality test was conducted to compare the variance explained in proteins and SBP by SNPs and to test their causal direction. All MR analyses were performed using the R package "TwoSampleMR".

There may be bias due to the participant overlap between the two GWAS studies [10], as both GWAS included participants from the UK Biobank. To assess this potential bias, we re-performed MR analysis based on the above harmonized datasets using R package "MendelianRandomization". For proteins with only 1 SNP instrument, Wald ratio was calculated. For proteins with 2 or more SNPs, inverse variance weighted MR with fixed effects was performed and if the heterogeneity test was significant, inverse variance weighted MR with random effects was performed. All the analyses were carried out using the function "MendelianRandomization::mr_mr_ivw", allowing setting a correlation parameter psi, which indicates the correlation between the association with the exposure and the association with the outcome for each variant resulting from sample overlap [11]. The parameter psi is set to 0 when there is no participant overlap, and arises if the samples for the associations with the exposure and the outcome overlap. Since we were unable to estimate the exact correction parameter psi among overlapping samples, we set several values for psi (0.1, 0.3, 0.5, 0.7, and 0.9) to assess the bias due to participant overlap.

All analyses were conducted by R version 4.1.0 (R Development Core Team, Vienna, Austria) and RStudio version 1.4.1717 (RStudio, Boston, MA, USA).

References

1. Holle R, Happich M, Lowel H, Wichmann HE, Group MKS. KORA--a research platform for population based health research. *Gesundheitswesen*. 2005;67 Suppl 1:S19-25. doi: 10.1055/s-2005-858235
2. Assarsson E, Lundberg M, Holmquist G, Bjorkesten J, Thorsen SB, Ekman D, Eriksson A, Rennel Dickens E, Ohlsson S, Edfeldt G, et al. Homogenous 96-plex PEA immunoassay exhibiting high sensitivity, specificity, and excellent scalability. *Plos One*. 2014;9:e95192. doi: 10.1371/journal.pone.0095192
3. Lin JS, Nano J, Petrera A, Hauck SM, Zeller T, Koenig W, Muller CL, Peters A, Thorand B. Proteomic profiling of longitudinal changes in kidney function among middle-aged and older men and women: the KORA S4/F4/FF4 study. *BMC Med*. 2023;21:245. doi: 10.1186/s12916-023-02962-z
4. Chalmers J, MacMahon S, Mancia G, Whitworth J, Beilin L, Hansson L, Neal B, Rodgers A, Ni Mhurchu C, Clark T. 1999 World Health Organization-International Society of Hypertension Guidelines for the management of hypertension. Guidelines sub-committee of the World Health Organization. *Clin Exp Hypertens*. 1999;21:1009-1060. doi: 10.3109/10641969909061028
5. Weuve J, Tchetgen Tchetgen EJ, Glymour MM, Beck TL, Aggarwal NT, Wilson RS, Evans DA, Mendes de Leon CF. Accounting for bias due to selective attrition: the example of smoking and cognitive decline. *Epidemiology*. 2012;23:119-128. doi: 10.1097/EDE.0b013e318230e861
6. Benjamini Y, Hochberg Y. Controlling the False Discovery Rate: A Practical and Powerful Approach to Multiple Testing. *J R Stat Soc Ser B-Stat Methodol*. 1995;57:289-300. doi: https://doi.org/10.1111/j.2517-6161.1995.tb02031.x
7. Sun BB, Chiou J, Traylor M, Benner C, Hsu YH, Richardson TG, Surendran P, Mahajan A, Robins C, Vasquez-Grinnell SG, et al. Plasma proteomic associations with genetics and health in the UK Biobank. *Nature*. 2023;622:329-338. doi: 10.1038/s41586-023-06592-6
8. Hemani G, Zheng J, Elsworth B, Wade KH, Haberland V, Baird D, Laurin C, Burgess S, Bowden J, Langdon R, et al. The MR-Base platform supports systematic causal inference across the human phenome. *Elife*. 2018;7. doi: 10.7554/eLife.34408
9. Evangelou E, Warren HR, Mosen-Ansorena D, Mifsud B, Pazoki R, Gao H, Ntritsos G, Dimou N, Cabrera CP, Karaman I, et al. Genetic analysis of over 1 million people identifies 535 new loci associated with blood pressure traits. *Nat Genet*. 2018;50:1412-1425. doi: 10.1038/s41588-018-0205-x
10. Burgess S, Davies NM, Thompson SG. Bias due to participant overlap in two-sample Mendelian randomization. *Genet Epidemiol*. 2016;40:597-608. doi: 10.1002/gepi.21998
11. Burgess S, Butterworth A, Thompson SG. Mendelian randomization analysis with multiple genetic variants using summarized data. *Genet Epidemiol*. 2013;37:658-665.

doi: 10.1002/gepi.21758

Figure S1. General study design of the MONICA/KORA study.
Abbreviations: KORA, Cooperative Health Research in the Region of Augsburg; MONICA, Monitoring of Trends and Determinants in Cardiovascular Diseases

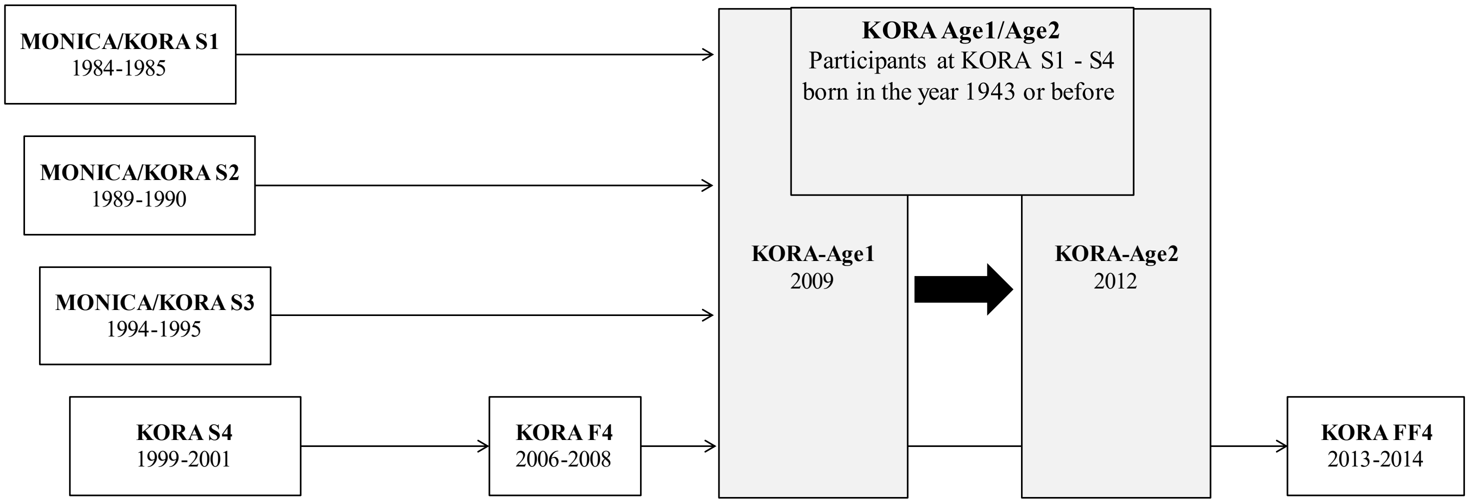

Figure S2. Pairwise correlation matrix between the 27 validated proteins associated with hypertension and systolic blood pressure (Figure 4B) among 1560 participants from the KORA S4 study.

Abbreviations: KORA, Cooperative Health Research in the Region of Augsburg; Full names of the proteins can be found in Table S1.

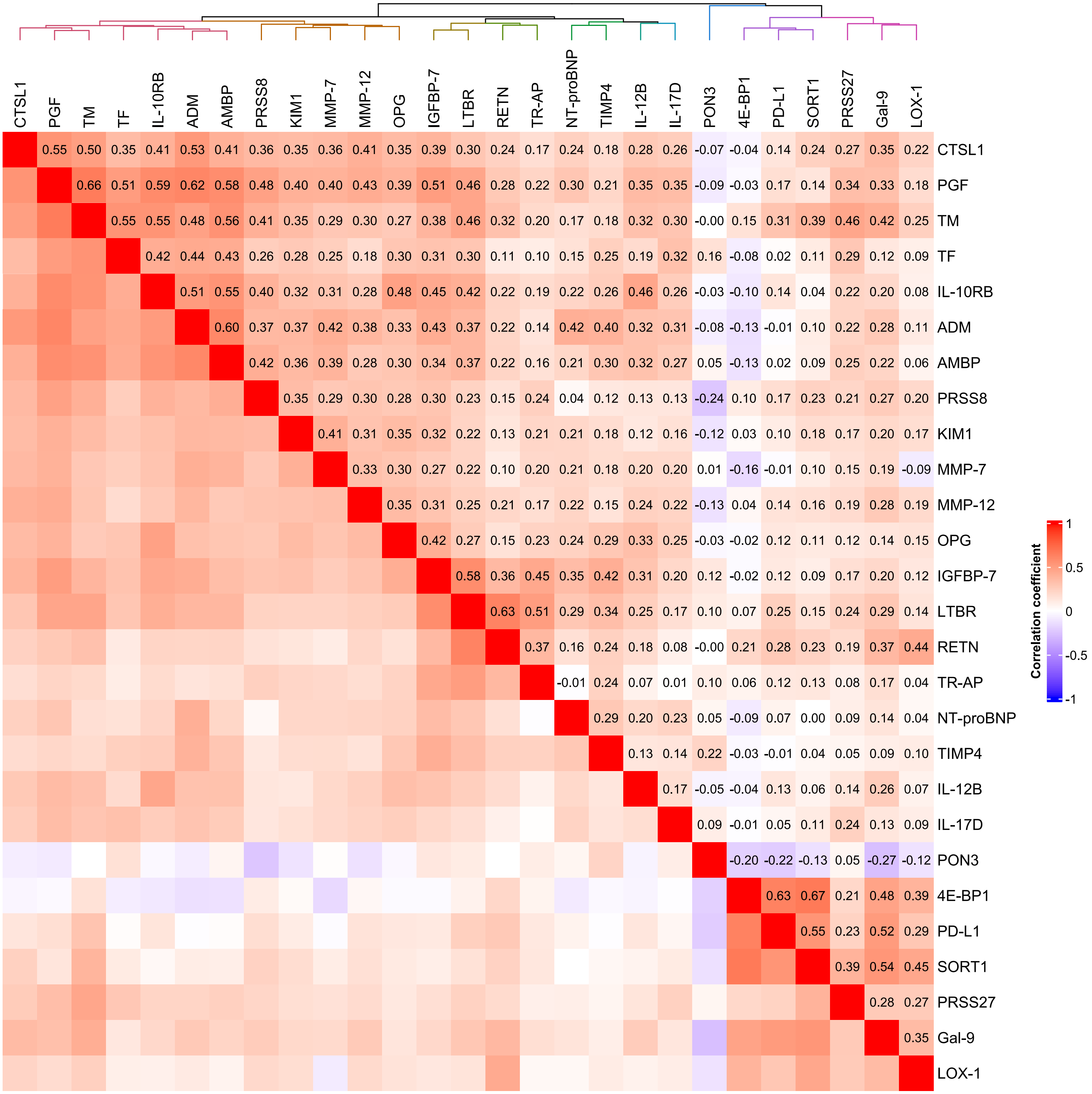

Figure S3. Pairwise correlation matrix between the 27 validated proteins associated with hypertension and systolic blood pressure (Figure 4B) among 1024 participants from the KORA-Age1 study.

Abbreviations: KORA, Cooperative Health Research in the Region of Augsburg; Full names of the proteins can be found in Table S1.

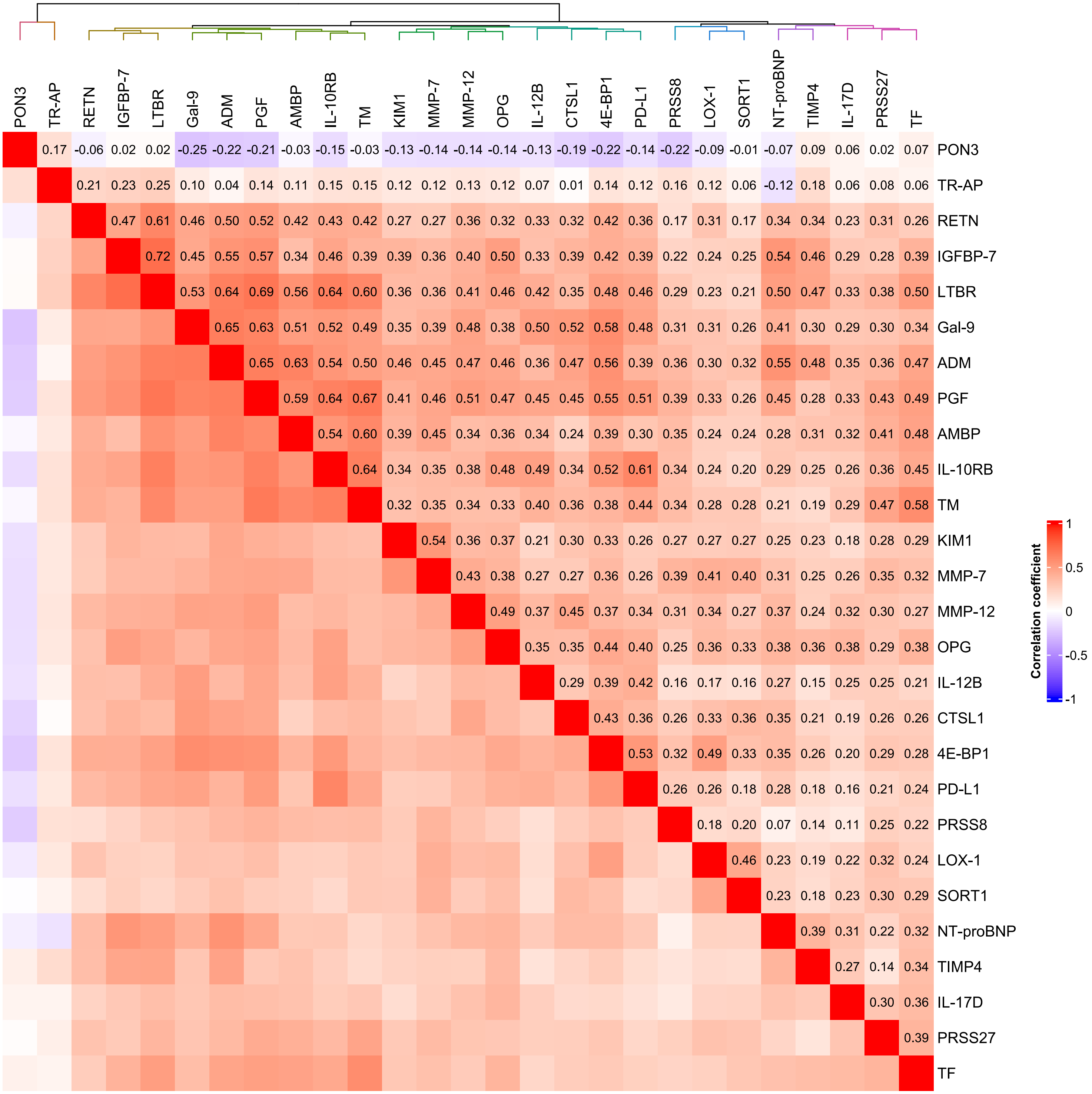

Supplement: Supplementary file 1 [file hyp-81-1156-s001.pdf]
